# Clinical factors associated with severe maternal outcomes in two South African hospitals: A case-control study

**DOI:** 10.1371/journal.pone.0346119

**Published:** 2026-04-15

**Authors:** Thwala Siphiwe Bridget Pearl, Blaauw Duane, Buchmann Eckhart, Ssengooba Freddie

**Affiliations:** 1 Centre for Health Policy, School of Public Health, University of the Witwatersrand, Johannesburg, South Africa; 2 Faculty of Health Sciences, University of Eswatini, Swaziland; 3 Department of Obstetrics and Gynaecology, University of the Witwatersrand, Johannesburg, South Africa; 4 School of Public Health, Faculty of Health Sciences, Makerere University, Kampala, Uganda; The University of Newcastle School of Medicine and Public Health, ETHIOPIA

## Abstract

**Background:**

A persistently high maternal mortality ratio (MMR) from direct pregnancy causes of maternal deaths in a district of the Gauteng Province prompted a detailed audit to identify clinical factors associated with severe maternal outcomes (SMOs).

**Methods:**

A cross-sectional matched case control study design was used to identify clinical factors associated with severe maternal outcomes in two regional hospitals in an urban district in Gauteng. 175 sequential SMO cases (near misses and maternal deaths) were matched to two different controls each. The first control was of women with complications while the second control was of women without complications. Data on individual and clinical obstetric characteristics of cases and controls was extracted from clinical case files. A Firth penalised conditional logistic regression was used to identify the clinical factors associated with severe maternal outcomes.

**Results:**

The overall SMO incidence ratio and MMR were 34.7 per 1000 live births and 130.8 per 100 000 live births respectively. Haemorrhage (48.8%) and hypertension (46.9%) were the most common underlying causes of SMOs. The SMO incidence ratio was 39.0 and 30.2 per 1000 live births for Hospital 1 and 2 respectively. Not receiving antenatal care was the most significant risk for SMOs (p < 0.001). In women with complications, not initiating ANC attendance (AOR = 11.0; CI = 2.6–46.0) or having less than 4 ANC visits (AOR = 2.2; CI = 1.2–4.2) had the highest risk for SMOs (p < 0.001). In women without complications, anaemia (AORs = 3.0; CI = 1.4–6.6; p < 0.01), and hypertension (AORs = 71; CI = 4.2−1,196.2), significantly increased the odds of SMOs.

**Conclusions:**

The severe maternal incidence was high in both hospitals. Obstetric haemorrhage and hypertension were significantly associated with most SMOs. Not attending ANC and having few ANC visits were important factors that showed significant association with poor maternal outcomes.

## Introduction

Emergency obstetric care (EmOC) is a set of interventions that have been proven effective in addressing the direct causes of maternal deaths [1,2]. However, unacceptably high maternal mortality persists in many countries [2]. Maternal near misses occur more frequently than maternal deaths, and near miss audits are useful for identifying factors that contribute to severe maternal outcomes (SMOs) [3,4]. Routine near miss audits have also been associated with reductions in maternal and neonatal deaths [5]. However, only few published near miss studies have included control groups in their design particularly in low and middle income countries [6–10]. The absence of a comparison group provides limited evidence that identified risk factors are actually responsible for near misses, and means that it is not possible to quantify the size of effects. Previous South African near miss studies [3,11–13] have also mainly been descriptive studies without controls.

It is encouraging that the MMR in South Africa has been declining in recent years, from a peak of 188 per 100 000 in 2009–134 per 100 000 in 2016 [14]. However, the rate remains very high at 111.7 per 100 000 compared to other middle-income countries [15]. The recent report of the National Committee for Confidential Enquiry into Maternal Deaths (NCCEMD) shows a rise in non-pregnancy related infections since the COVID-19 pandemic [15,16]. This trend seems to show an unfortunate reversal of previous gains in the reduction on non-pregnancy related infections of maternal deaths credited to the success of government initiatives to reduce maternal deaths from HIV. Meanwhile, mortality from direct pregnancy causes of maternal deaths has remained high as well [14]. This study therefore focuses on direct pregnancy causes of maternal deaths and was conducted before the COVID-19 pandemic. A number of clinical factors have been attributed to preventable maternal mortality in hospitals. These include antenatal care attendance, parity, presence or absence of disease (e.g., hypertension), and others [14]. Although these clinical factors are well documented, more data on the relative sizes of their effects would aid prioritisation for action, especially in settings with limited resources such as South Africa. This case-control study was nested in a broader cross-sectional research project investigating health system factors contributing to severe maternal outcomes and these are reported elsewhere [17,18]. In this paper, we sought to determine the near miss incidence ratio, maternal mortality ratio (MMR), severe maternal outcome ratio (near misses and deaths), and correlations of obstetric factors to SMOs in two South African regional hospitals in the Gauteng Province. We also explored what the most sensitive control would be for showing predictors for severe maternal outcomes between an uncomplicated control and control with mild or moderate complications.

## Materials and methods

A matched case control study was conducted from the 1^st^ of June 2015–31^st^ July 2016 in two public regional hospitals in an urban district of the Gauteng Province. This district had an MMR of 169 per 100 000 live births at the time of the study, higher than the provincial average of 113 per 100 000 live births [19]. The two hospitals collectively delivered over 20 000 live births annually. They acted as referral hospitals for district hospitals and community health centres (CHCs), and therefore had medical specialists including obstetricians, intensive care units, laboratories and blood banks.

The two hospitals were selected to provide a comparison analysis of health system factors that could potentially explain their differences in performance (e.g., maternal mortality) as they were both level II, government funded, and located in the same district. Selection of study sites was done collaboratively with the Gauteng Department of Health. The results from this analysis are reported elsewhere [18,20]. The two level II hospitals were purposively selected in one district of the Gauteng Province based on outcome maternal indicators such the numbers of maternal deaths, perinatal deaths, annual births; willingness of the hospitals to participate in the audit; and approval of provincial and district health authorities. A poorer performing public regional hospital (hospital 1) was selected, and compared to a better performing public regional hospital (hospital 2). The better performing hospital was the oldest regional hospital in the district, and located in an industrial town that serviced high proportions of economically disadvantaged women from surrounding townships. It had a catchment area of about 1.2 million people [21], and had approximately 540 hospital beds. The poorer performing hospital was also a regional hospital that had been recently constructed in a township of the same district, and had 821 beds. It serviced three neighbouring townships as well. Both hospitals had high volumes of women requiring childbirth services including EmOC.

### Cases and controls

Following prospective surveillance of all deliveries during the broader cross-sectional study period, cases and corresponding controls were selected. SMOs (composed of near-misses and maternal deaths) in the hospitals were defined as cases. An obstetric near-miss was defined as a woman with acute organ dysfunction or failure during pregnancy, labour, or up to 48 days postpartum in keeping with World Health Organisation (WHO) criteria [22], as summarised in S1 Table. WHO uses a combination of laboratory, clinical, and management criteria as the basis for identification of a near miss [22]. The WHO criteria were adjusted to include eclampsia, uterine rupture as recommended for low-and middle-income country (LMIC) settings [23]. Because of the scarcity of blood in many South African hospitals, where women in need of blood often received less than they needed or sometimes did not get any, we adjusted the WHO definition of 5 units to define a near miss to at least 1 unit of blood received. Typically, only very seriously ill women (such as maternal near misses) tend to have whole blood prescribed for therapy. This adjustments in the SMO definition have been done according the near miss criteria first done in Haydom in Ethiopia [24] and has also been applied in near miss studies conducted in Rwanda [6] and Tanzania [23,24].

Two different controls were matched to every case. The first control was a woman without any complications who progressed to a normal vaginal delivery. The second control was a woman with similar complications to the case, but who did not progress to the level of a near miss or a maternal death. Complications were defined as any difficulties in the pregnancy and or delivery. These included bleeding of at least 500mls or 1000mls for normal vaginal deliveries and Caesarean section deliveries respectively; any hypertension during the current pregnancy, delivery or postpartum; antenatal or postpartum clinical infection that triggered a systemic inflammatory response (septicaemia); and abortions (termination before 20 weeks gestation). We also matched both controls by age, parity, and mode of delivery (e.g., assisted vaginal delivery). Age categories were < 18 years, 18–34 years, and>=35 years. Parity categories were; Para 0, 1–4, and>=5.

### Sampling strategy and size

We estimated the sample size for health facilities in the whole district based upon the WHO established prevalence of 7.5 near misses per 1000 live births [25]. The required sample size for the matched case-control study was calculated as 165 cases matched to two controls, assuming an odds ratio of 2, power of 80%, probability of exposure 0.75 in cases, alpha of 0.05. An additional 5% were added for a final desirable sample size of about 173 triplets (case plus two controls). By the end of the study period, a total of 175 cases were included and successfully matched to women with uncomplicated deliveries. However, only 140 cases could be matched to women with similar complications because of the low frequency for some maternal conditions (e.g., epilepsy in pregnancy).

**Fig 1:**
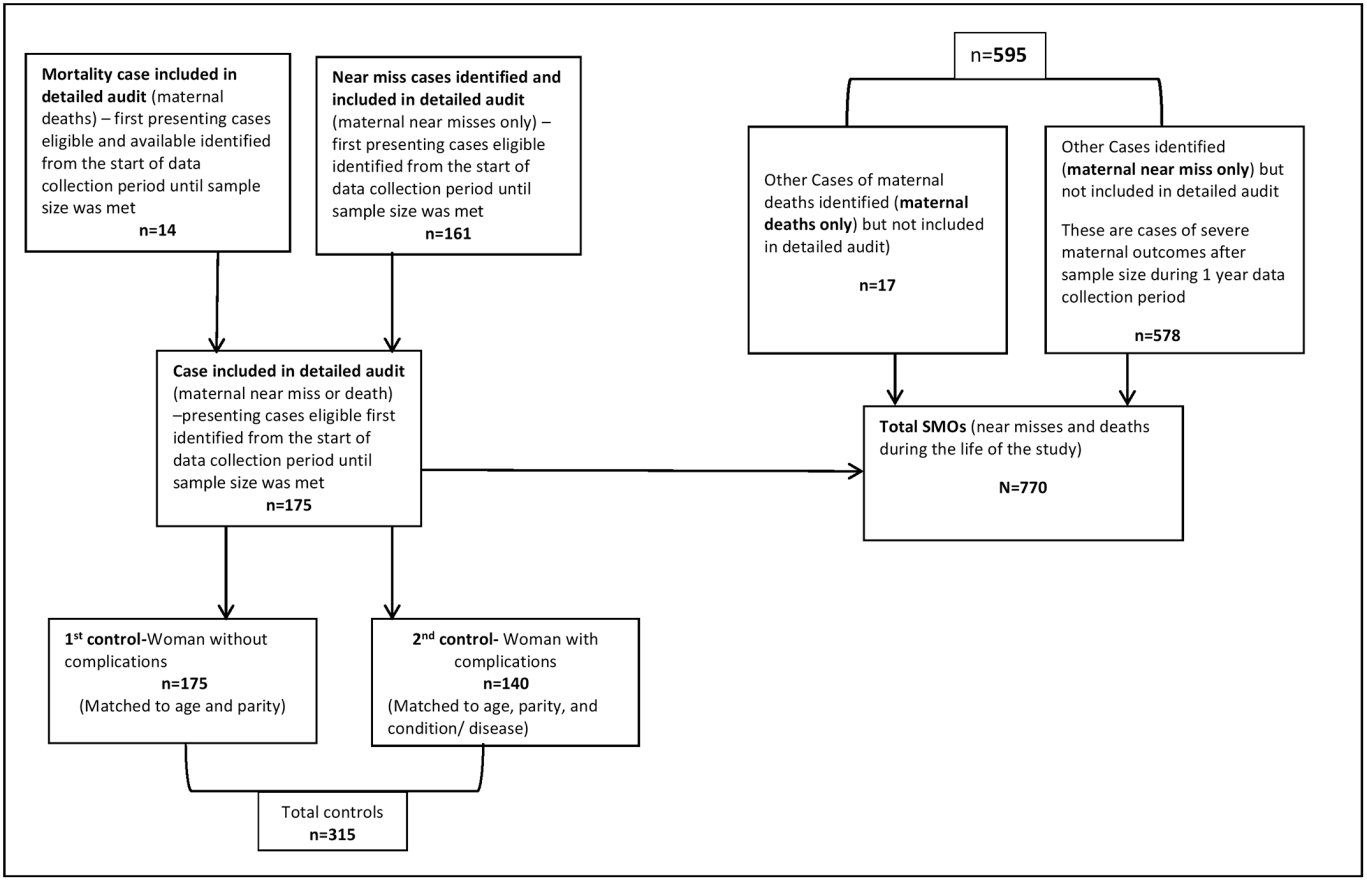
Sample selection flow chart.

Throughout the 12 month study surveillance period, a total of 739 near miss cases were identified and 30 maternal deaths occurred in the study hospitals. For the detailed audit 175 prospective cases were enrolled for the detailed audit to fulfil the required sample size during the same time period. The first 175 presenting cases were selected consecutively over a period of 12 months. Sequential rather than simple random sampling was necessary to complete the prospective enrolment of cases and controls within a reasonable time period. Because this included all eligible cases over a fairly long period, and we do not expect significant seasonal variation in the contributors to near miss, this strategy should not have introduced bias in the final sample. For every case identified, the first presenting suitable matched controls were selected. Selecting the first control (a woman without complications) was relatively quicker as women without complications occurred commonly. The second control took more time because that required matching by complication (Fig 1).

### Recruitment and data collection

On alternate days in each hospital, study researchers went through patient registers all labour, antenatal, postnatal, gynaecology wards, as well in intensive care units (ICUs), to identify cases of maternal deaths and near misses. We also used maternity clinical heads, nurse managers and doctors in the study hospitals to help identify potential cases. In addition, researchers attended the weekly maternal mortality and morbidity meetings which discussed adverse events and all cases of severe morbidity or mortality of women and new-borns that would have occurred the past month.

Controls (women without complications and women with complications) for each enrolled case were retrospectively and systematically selected at least 48 hours after the case had been identified. Controls were chosen by selecting the first woman presenting without a complication that matched the case (control 1) and the first presenting eligible woman with a complication that occurred and matched the case (control 2). The researchers located the clinical files of cases and controls from the patient file archives as the patients had already been discharged or transferred by the time of data collection onto audit tools.

The South African confidential enquiries into maternal deaths tool was adapted and used to extract data from retrieved patient files [26]. The data collection tool included study category (case, control1, control2), near-miss criteria, demographic characteristics, obstetric history, antenatal history, medical history, HIV status, obstetric and/or medical complications, key interventions (e.g., blood transfusion, ICU admission), delivery details, and pregnancy outcomes. Data were collected by the primary researcher (a research midwife) supported by two trained fieldworkers. The primary researcher also checked all audit tools for completeness, created and maintained a database to keep track of groups (cases and corresponding controls), and processed audit tools for data entry into Microsoft Excel software.

We reviewed each case with hospital teams during mortality and morbidity meetings to determine causes of death or near-miss, as well as discuss the circumstances around each case and their impact on outcomes. An independent obstetrician consultant reviewed a sample of cases (n = 28) and made judgements about cause of death based on case notes, and completed data collection forms. These were then compared with those completed by the researcher to establish consistency in conclusions. Overall, judgements on causality were consistent except in two cases (7.1%), where the consultant’s opinion were deemed superior and adopted.

### Data analysis

Data were captured using Microsoft Excel software and imported into Stata14 for further processing and analysis. Statistical differences between cases and corresponding controls were compared using matched case-control analysis. Penalised conditional logistic regression was used to evaluate the association of predictors on the likelihood of being a case rather a control, with separate analyses for the two different controls. Logistic analysis used for binary outcomes always requires relative comparisons. This produces difficulties when the outcome occurs in the cases but is found very rarely or not at all in the comparison groups. These are important large effects that cannot be ignored. Firth penalised logistic regression is the recommended statistical approach for dealing with such data separation [27–29]. A 20% significance level in bivariate analysis was used as the criteria for initial inclusion in the development of the multiple regression models. Adjusted odds ratios (AOR) at 95% confidence interval (CI) and a 5% significance level were considered in the final multivariate model.

Table 3 shows the breakdown of each identified factor for controls and cases, as well as the odds ratio and p-values from the Firth conditional logit analysis. Some complications were uncommon in the controls producing large odds ratios as would be expected for such large differences. However, the confidence intervals are wide despite the Firth correction because of the small numbers. Potential effects of age, parity and hospital are not estimable in this analysis because cases and controls were matched on those variables. We also considered whether routine and emergency obstetric care was given to women as per the South African maternal health guidelines [30]. Furthermore, routine and emergency care given by health providers to manage women was also observed and measured against South African Maternal Health Guidelines [31] (S2 Table).

**Table 3:** Demographic factors, obstetric predictors and neonatal outcomes of SMO cases.

			SMOs compared to Uncomplicated controls (n=175)				SMOs compared to Complicated controls (n=140)			
			Controls	Cases	OR (95% CI)	p-value	Controls	Cases	OR (95% CI)	p-value
**Demographic factors**	**Marital status**	Not single	82 (50.0%)	104(62.7%)	**0.58 (0.36 – 0.92)**	**0.020**	69 (53.5%)	80 (60.6%)	0.66 (0.39 – 1.11)	0.117
		Single	82 (50.0%)	62(37.3)			60 (46.5%)	52 (39.4%)		
**Obstetric factors**	**ANC attended**	Un-booked	5 (2.9%)	21 (12.0%)	**5.57 (2.01 – 21.03)**	**<0.001**	2 (1.4%)	19 (13.6%)	**35.0 (4.78 – 4459.9)**	**<0.001**
	**(Initiating ANC status)**		170 (97.1%)	154 (88.0%)			138 (98.6%)	121 (86.4%)		
	**Timing of initiating ANC**	<20 weeks	65 (40.4%)	47 (36.2%)	1.42 (0.90 – 2.26)	0.139	54 (41.5 %)	41 (37.6%)	1.46 (0.88 – 2.44)	0.142
		>=20 weeks	96 (59.6%)	128 (63.9%)			76 (58.5%)	68 (62.4%)		
	**Number of ANC visits**	<4 visits	51 (21.9%)	69 (39.4%)	**0.45 (0.29 – 0.69)**	**<0.001**	42 (30.0%)	55 (39.3%)	**0. 41 (0.25 – 0.67)**	**<0.001**
		>=4 visits	124 (70.9%)	106 (60.6%)			98 (70.0%)	85 (60.7%)		
	**Risks during ANC**	No	102 (58.3%)	67 (38.3%)	**2.43 (1.54 – 3.96)**	**<0.001**	51 (36.4%)	55 (39.3%)	0.87 (0.51 – 1.46)	0.596
		Yes	73 (41.7%)	108 (61.7%)			89 (63.6%)	85 (60.7%)		
	**Referred into the hospital**	No	142 (81.1%)	128 (73.1%)	1.6 (0.96 – 2.7)	0.072	90 (64.3%)	96 (68.6%)	0.82 (0.49 – 1.36)	0.442
		Yes	33 (18.9%)	47 (26.9%)			50 (35.7%)	44 (31.4%)		
	**Previous C/S**	No	168 (96.0%)	147 (84.0%)	**4.23 (1.91 – 10.90**	**0.001**	109 (77.9%)	115 (82.1%)	0.66 (0.30 – 1.37)	0.263
		Yes	7 (4.0%)	28 (16.0%)			31 (22.1%)	25 (17.9%)		
	**HIV positive**	Negative	129 (73.7%)	123 (70.3%)	1.19 (0.74 – 1.92)	0.470	93 (66.4%)	100 (71.4%)	0.77 (0.45 – 1.32)	0.340
		Positive	46 (26.3%)	52 (29.7%)			47 (33.6%)	40 (28.6%)		
	**Adverse medical history**	No	175 (98.9%)	171 (97.7%)	1.80 (0.40 – 10.32)	0.447	136 (97.1%)	136 (97.1%)	1.00 (0.26 – 3.87)	1.000
		Yes	2 (1.1%)	4 (2.3%)			4 (2.9%)	4 (2.9%)		
	**Prolonged labour**	No	175 (100.0%)	169 (96.6%)	**13.0 (1.54 – 1695.8)**	**0.014**	136 (97.1%)	134 (95.7%)	1.44 (0.44 – 5.22)	0.545
		Yes	0 (0.0%)	6 (3.4%)			4 (2.9%)	6 (4.3%)		
	**Multiple pregnancy**	No	175 (100.0%)	169 (96.6%)	**13.0 (1.54 – 1695.8)**	**0.014**	135 (96.4%)	134 (95.7%)	1.18 (0.38 – 3.87)	0.773
		Yes	0 (0.0%)	6 (3.4%)			5 (3.6%)	6 (4.3%)		
	**Anaemia**	No	163 (93.1%)	145 (82.9%)	**2.9 (1.44 – 6.39)**	**0.003**	123 (87.9%)	117 (83.6%)	1.39 (0.73 – 2.71)	0.320
		Yes	12 (6.9%)	30 (17.1%)			17 (12.1%)	23 (16.4%)		
	**Syphilis**	Negative	174 (99.4%)	171 (97.7%)	7.00 (0.68 – 941.2)	0.112	137 (97.9%)	138 (98.6%)	0.71 (0.12 – 3.67)	0.680
		Positive	1 (0.6%)	4 (2.3%)			3 (2.1%)	2 (1.4%)		
	**Proteinuria**	No	175 (100.0%)	171 (97.7%)	9.0 (0.96 – 1192.6)	0.055	138 (98.6%)	136 (97.1%)	1.80 (0.40 – 10.32)	0.447
		Yes	0 (0.0%)	4 (2.3%)			2 (1.4%)	4 (2.9%)		
	**Hypertension**	No	175 (100.0%)	148 (84.6%)	**55.0 (7.73 – 6973.0)**	**<0.001**	105 (75.0%)	115 (82.1%)	0.57 (0.29 – 1.11)	0.098
		Yes	0 (0.0%)	27 (15.4%)			35 (25.0%)	25 (17.9%)		
	**Pre-eclampsia**	No	175 (100.00%)	119 (68.00%)	**165.9 (23.5 – 21 016.8)**	**<0.001**	109 (77.9%)	89 (63.6%)	**4.08 (1.84 – 10.5)**	**<0.001**
		Yes	0 (0.0%)	56 (32.00%)			31 (22.1%)	51 (36.4%)		
**Outcomes**	**Apgar 1 minute**	7-10	164 (95.9%)	99 (66.9%)	**–**	–	114 (85.7%)	81 (66.9%)	–	–
		4-6	5 (2.9%)	26 (17.6%)	**7.97 (3.30 – 22.93)**	**<0.001**	14 (10.5%)	21 (17.4%)	**3.15 (1.38 – 8.27)**	**0.006**
		0-3	2 (1.2%)	23 (15.5%)	**8.26 (1.77 – 79.20)**	**0.027**	5 (3.8%)	19 (15.7%)	**7.25 (1.26 – 79.15)**	**0.025**
	**Apgar 5 minutes**	7-10	169 (98.8%)	120 (81.1%)	–	–	123 (94.6%)	97 (80.2%)	–	–
		4-6	1 (0.6%)	7 (4.7%)	**7.03 (1.51 – 67.30)**	**0.041**	3 (2.3%)	7 (5.8%)	**4.82 (1.08 – 44.46)**	**0.038**
		0-3	1 (0.6%)	21 (14.2%)	**15.5 (1.73 – 2 038.4)**	**<0.001**	4 (3.1%)	17 (14.1%)	**15.73 (1.12 – 2 570.7)**	**0.039**
	**Low birth weight**	No	141 (89.8%)	74 (64.4%)	**5.6 (2.61 – 14.24)**	**<0.001**	94 (79.0%)	58 (63.0%)	2.07 (0.89 – 5.24)	0.092
	(<2500g)	Yes	16 (10.2%)	41 (35.7%)			25 (21.0%)	34 (37.0%)		

OR,CI and p value *from* Firth conditional logistic regression, p value<0.05 *in* bold

### Ethics

Ethical clearance to conduct this study was obtained from the University of the Witwatersrand Human Scientific Research Ethics Committee-Medical (Clearance certificate No: M130967) on the 5^th^ October 2013 and the Ekurhuleni Health District Research Committee (Project certificate no: 24/02/2015–1) on the 25^th^ February 2015. The Gauteng Province and the two study hospitals also gave permission for the study. Participants were given information about the study before extending an invitation for participation. They were also given information sheets for their reference with contact details in case they wished to obtain clarity or had follow-up questions. Written informed consent was obtained from participants. The hospitals however granted permission for patient file review, and this was approved by ethics committees in the district and the university. Identifying characteristics of participant (e.g., name and address) were not recorded onto data collection forms. Patient records were not anonymised before being accessed by researchers. However, researchers were bound by confidentiality clauses as part of the ethics application process not to disclose or discuss medical records information or patient identifying characteristics. Neither were participant patient files duplicated by researchers or removed from hospital archives. Identifying participant characteristics were not reported during dissemination meeting, reports, or scientific communication.

## Results

### Maternal outcome indicators

A total of 739 near-misses and 31 maternal deaths were identified (Table 1) between 15 June 2015 and 31July 2016. There were 22174 live births during this study period. Therefore, the overall SMO ratio (maternal deaths plus near-misses) was 34.7 per 1000 live births (Table 1). The near-miss incidence ratio was 33.33 per 1000 live births. The combined hospital/ institutional maternal mortality ratio (iMMR) for the two hospitals was high at 140 per 100 000 live births. Hospital1 contributed the most to mortality in this study with an iMMR of 230 per 100 000 live births as compared to 46 per 100 000 live births in Hospital2 (Table 1).

**Table 1:** Maternal outcomes in study hospitals during study period.

Indicator	Total	Hospital 1	Hospital 2
Total live births	22174 (100.0%)	11315 (51.0%)	10859 (49.0%)
Total near-misses	739 (100.0%)	416 (56.3%)	323 (43.7%)
Total maternal deaths	31 (100.0%)	26 (83.9%)	5 (16.1%)
Near miss incidence ratio	33.3 per 1000 live births	36.8 per 1000	29.7 per 1000
Severe maternal outcome (SMO) ratio:	34.7 per 1000 live births	39.0 per 1000	30.2 per 1000
Mortality index	3.2 per 100	3.8 per 100	0.9 per 100
Hospital Maternal mortality ratio (iMMR)	139.8 per 100 000 live births	229.8 per 100 000	46.0 per 100 000

### Underlying causes of SMO

Table 2 shows that the leading underlying causes for near-miss (n = 161) were obstetric haemorrhage (49%), and hypertension (47%). Anaemia was a noteworthy contributing factor (21%). Some women experienced near miss events due to prolonged labour (4.4%), abortion (3.8%), and sepsis (3.1%). Indirect underlying causes for near misses included HIV (1.3%), cardiac problems (1.3%), and epilepsy (0.6%). Maternal deaths (n = 14) were attributed to obstetric haemorrhage (43%), sepsis (21.4%), abortion (14.3%), embolism (14.3%), and HIV (28.6%). Deaths from hypertension (7.1%) and trophoblastic disease (7.1%) were less common.

**Table 2:** Underlying causes for SMOs in cases selected for the detailed audit.

Underlying causes for near misses (n = 161)	Frequency
Anaemia (contributory)	33 (20.6%)
Hypertension	75 (46.9%)
Obstetric haemorrhage	78 (48.8%)
Prolonged labour	7 (4.4%)
Abortion	6(3.8%)
Ectopic pregnancy	2 (1.3%)
Sepsis	5 (3.1%)
HIV	2 (1.3%)
Pulmonary oedema	1 (0.63%)
Cardiac problems	2 (1.3%)
Epilepsy	1 (0.6%)
**Underlying causes for maternal deaths (n = 14)**	**Frequency**
Obstetric haemorrhage	6(42.9%)
HIV	4 (28.6%)
Sepsis	3 (21.4%)
Abortion	2 (14.3%)
Embolism	2 (14.3%)
Trophoblastic disease	1 (7.1%)
Hypertension	1 (7.1%)

### Demographic and clinical predictors- bivariate analysis (odds ratios)

Results of the bivariate analysis of socio-demographic and clinical predictors of being an SMO (case), in comparison with women without or with obstetric complications (controls), are shown in Table 3. Not receiving ANC was significantly more likely to result in severe maternal outcomes for both control comparisons. Having 4 or more ANC visits was protective against SMOs. In the control group of women without complications, having a previous Caesarean section (p = 0.001); prolonged labour (p = 0.014); multiple pregnancy (p = 0.014); anaemia (p = 0.003); significantly increased the odds for severe maternal outcomes. Hypertensive disease was highly likely to result in severe maternal outcomes and this was equally significant in both control groups (p = 0.001). The odds for severe outcomes were higher in the control group without complications however. These results show that the effect of independent factors on outcomes was muted in the control group of women with complications while the effect was clearer in the control group of women without complications. This suggests that not matching the disease / condition in a control group makes it more sensitive when measuring the relationship between independent variables (factors) and outcomes (severe maternal outcomes). Furthermore, the likelihood for babies to be born with poor Apgar’s were significantly higher in cases than in both controls. Table 3 illustrates.

### Women without complications

Having a partner present (not being single) was protective of women against SMOs (OR=0.58; CI = 0.36–0.92; P = 0.02). Having a previous Caesar, multiple pregnancy, prolonged labour, syphilis, and hypertension all increased the likelihood for SMOs significantly (p < 0.05). Anaemia was an important contributor for SMOs and increased the likelihood nearly 3 times (OR=2.9; 95%CI = 1.44–6.39; p < 0.003). Table 3 illustrates.

### Women with complications

Not initiating ANC was more likely to result in a SMO (OR= 35; 95%CI = 4.78–4459.9; p < 001). Where women booked ANC and had more than 4 ANC visits, SMOs were significantly less likely to occur (OR=0.41; CI = 0.25–0.67; p < 0.00). Pre-eclampsia increased the risk for SMOs at least 4 fold (CI = 1.84–10.5) and this was significant (p < 0.00). This is shown in Table 3.

### Multiple regression analysis- adjusted odds ratios (AORs)

If ANC attendance was not initiated, women without complications and women with complications had the odds of at least 9 and 11 respectively for developing SMOs and this was highly significant (p < 0.00). Having less than 4 ANC visits significantly increased the odds at least 2 fold in both groups. In women without complications, hypertension had significant risks for SMOs (AOR = 70.97; 95%CI = 4.21–1196.16; p = 0.003) and anaemia was an important contributor (AOR = 3.04; 95%CI = 1.39–6.64; p = 0.005). In women with complications however, hypertension and anaemia also increased the risks for SMOs but had lower significance as seen in Table 4.

**Table 4:** Multiple regressions: Predictors of SMO.

	SMO compared to Uncomplicated controls			SMO compared to Complicated controls		
	OR	CI	p-value	OR	CI	p-value
**Multiple pregnancy**	20.57	(1.14 - 372.60)	0.041 *	2.20	(0.62 - 7.78)	0.222
**ANC attendance**						
< 20 weeks, ≥ 4 visits	–			–	–	
≥ 20 weeks, ≥ 4 visits	1.18	(0.60 - 2.32)	0.635	1.09	(0.55 - 2.17)	0.807
< 4 visits	2.00	(1.06 - 3.79)	0.032 *	2.21	(1.15 - 4.24)	0.017 *
Un-booked	9.61	(3.25 - 28.39)	<0.001 ***	11.00	(2.63 - 46.01)	0.001 **
**Anaemia**	3.04	(1.39 - 6.64)	0.005 **	1.60	(0.77 - 3.31)	0.206
**Hypertension**	70.97	(4.21 − 1,196.16)	0.003 **	0.75	(0.40 - 1.40)	0.360
**Prolonged labour**	16.39	(0.75 - 358.42)	0.076	1.73	(0.41 - 7.20)	0.454
**HIV positive**	1.31	(0.73 - 2.33)	0.366	0.85	(0.48 - 1.49)	0.568

*Firth conditional logistic regression*

*** p < 0.001, ** p < 0.01, * p < 0.05

### Quality of care process indicators

S2 Table shows oxytocin as the drug most commonly used routinely (98.6%) to prevent postpartum haemorrhage as per national guidelines [31]. But only about 61% of women in labour had the partogram used to monitor their labour showing a sub-optimal adherence to standards of practice detailed in the South African national maternal guidelines [30]. All women that delivered by caesarean section received prophylactic antibiotics as per the guidelines [30,32]. However, only two thirds (66.67%) of women with preeclampsia were given magnesium sulphate, contrary to maternal health guideline [30] prescriptions.

## Discussion

The study found that the study hospitals had high incidences of poor maternal outcomes and this was consistent with the overall district trend as evidenced by high MMR reported by the NCCEMD [14]. The overall incidence for SMOs (near-misses and deaths) in this study was 34.7 per 1000 live births. Overall, the maternal mortality index was 3.2 per 100. The overall hospital iMMR was high at 131 per 100 000, and this was consistent with the district MMR [33,34]. Hospital1 had a much higher mortality index (3.78 per 100) compared to Hospital2 (0.92 per 100) implying that women with SMOs were nearly 4 times more likely to die in Hospital1 compared to Hospital2. The population serviced by Hospital1 is in the city while the population serviced by Hospital2 is in the township, characterised by poorer socio-economic status compared to the city population. It has been shown that populations from better socio-economic backgrounds (e.g., urban) tend to have better outcomes than those from poorer socio-economic backgrounds [35]. Differences in the maternal outcomes of the two hospitals, although similarly resourced by the government, in part, suggest that differences in the profiles of the populations they service has bearing on health outcomes. In South Africa, township populations tend to have even lower socio-economic demographics than city populations [36,37].

Haemorrhage (49%) and hypertension (47%) were the leading direct causes of near miss cases and this mirrors findings from other near-miss studies [3,38–42] as well as the South African NCCEMD reports [14,33]. The presence of hypertension increased the odds for SMOs considerably in women without complications. Anaemia was also an important contributory factor for SMOs in women with complications (AOR = 3.04; 95%CI = 1.39–6.64; p < 0.001). Meanwhile, not receiving ANC exposed women without complications (AOR = 9.6; 95%CI = 3.25–28.39; p < 0.001) and women with complications (AOR = 11; 95%CI = 2.63–46.01; p = 001) to considerably more SMOs.This study found hypertension in childbearing women to have increased risk of SMOs. It supports literature in showing the dangers of hypertension in pregnancy to mothers and their babies [43,44]. Included in the ANC package is screening for hypertension in pregnancy, with an intention to control it as soon as it manifests, to minimise its effect on the pregnancy [30,45]. In South Africa, The NCCEMD had expressed concern that mortalities specific for hypertension in pregnancy have remained unyieldingly high, even though deaths from the other co-leading causes (haemorrhage and HIV) are slowly declining [14,46]. The 2023 NCCEMD report still shows hypertension leading haemorrhage as a direct cause for maternal death [15]. Pre-eclampsia is a serious pregnancy complication that results in SMOs [47–49]. Magnesium sulphate is an effective treatment, and is the recommended first line drug of choice to prevent convulsions in severely pre-eclamptic women [50–52]. In this study however, we observed that as much as 40% of women with pre-eclampsia did not receive magnesium sulphate to prevent eclampsia, despite clear national maternal guidelines recommending its use [31]. It is concerning that not all pre-eclamptic women got magnesium sulphate despite clear prescriptions by maternal health guidelines of the country. This was an important gap in quality of care despite clear policy guiding its use. So was the non-use of partograms for women in labour. Partogram use is important to prevent prolonged labour, a significant complication linked to poor maternal and neonatal outcomes [53–55] Further empirical inquiry is required to explore implementation barriers to of these clinical guidelines. These findings were intended to inform the Gauteng Province when planning for clinical strategies to improve maternal outcomes in the public hospitals of the district. To improve outcomes, strategies should prioritise interventions to manage these conditions to reduce their impact.

It is understood that attending ANC during pregnancy is important to allow for screening of both medical and pregnancy related morbidities [45,56,57]. This study confirms that women that do not attend ANC have a significantly greater risk of SMOs than those that do attend ANC during their pregnancies. Although the effective coverage of ANC services has improved through health system interventions to improve access, such as the abolition of user fees for women [58], utilisation of ANC is not yet absolute. Knowledge of the benefits of ANC has long been attributed to utilisation of the service [59]. Therefore, health education of women on the benefits of ANC attendance should be sustained. In addition, the frequency of ANC is also important to assure positive outcomes of the pregnancy. We found that the higher the number of ANC visits in a pregnancy, the less the likelihood of SMOs. The latest WHO recommendations for ANC insist on at least 8 ANC visits during a woman’s pregnancy [45]. Studies on utilisation of ANC show that women care about the quality of care they receive. Careful attention is needed of the quality of ANC as determined by women to improve the frequency of ANC visits and support good maternal outcomes. A meta-analysis of qualitative evidence globally also showed that women expectations of care went beyond health procedures and tests during ANC [60]. Women were looking for quality ANC that promoted a positive pregnancy experience [61]. Timely information along with psychosocial support of women by health providers were missing domains needed in ANC to encourage women to attend [60]. Amnesty International added that transport costs, poor attitudes of health workers, and lack of privacy in public clinics in the two rural provinces were deterrents to ANC attendance for South African women [62]. All these will have to be attended to in order to encourage women to attend ANC more frequently in their pregnancies.

Anaemia was a strong contributor to SMOs in this study. It is also a known enabler for many detrimental conditions perinatally (e.g., postpartum haemorrhage) in the literature [63,64]. The effect of anaemia in pregnancy is dire on both maternal and neonatal outcomes. If uncorrected in time during pregnancy, anaemia places women a risk for infection and potentially sepsis and pre-eclampsia [65]. In addition, women with a normal haemoglobin at the time of labour and delivery tend to fare better when faced with obstetric haemorrhage [66]. Efforts to resolve anaemia during the pregnancy should therefore be promoted and intensified as far as possible. The presence of anaemia during pregnancy and delivery also increases the likelihood of complications with haemorrhage postpartum [67–69]. Severe anaemia is particularly predictive of uterine atony and the performance of hysterectomies [67]. In this study haemorrhage was also identified as a factor responsible for severe maternal outcomes. It is therefore concerning that anaemia was prevalent in this study and may in part explain, the high incidence of obstetric haemorrhage observed. In agreement Maswime and Buchmann observed that pre-operative anaemia was an associated risk factor for haemorrhage in at least 55% of maternal near misses [70]. In a systematic inquiry including all geographic economic regions of the world, Daru and colleagues observed that severe anaemia was still an important indirect cause of maternal mortality [71]. They proposed that resolving anaemia in pregnant women should be maintained as a health priority worldwide and in the research agenda [71]. WHO recommends prophylaxis for anaemia in all pregnant women, and treatment to prevent morbidity and mortality of child bearing women [45]. The South African government also upholds routine screening, and prophylactic treatment of pregnant women from anaemia in ANC [30,72]. However, evidence from this study implies that anaemia is still a significant risk in child bearing woman in this South Africa population. Efforts put by the SA government to prevent and treat anaemia in pregnant women needs to be upheld and even increased as far as possible to improve maternal outcomes.

### Strengths and limitations

Few studies have included a comparison group in the current near-miss literature, a missed opportunity particularly in LMIC settings. The use of controls in near miss studies affords more rigorous exploration of the relationship between postulated contributors and maternal outcomes. The quantitative analysis makes it possible to rank factors by importance, thereby enabling prioritisation during planning. In this study for example, we were able to quantify the importance of ANC attendance in preventing SMOs. The use of two different types of controls allowed us to measure the size of the effect of independent factors to outcomes when women have no complications or when they have them. Two different types of controls were also used to see if there would be differences in the health system factors contributing to severe maternal outcomes. This analysis is reported elsewhere [20]. From a methods and analysis perspective, we learnt that unmatched controls were stronger in showing the size of the effect between contributors and outcomes.

Restricted access to some files of maternal death patients (e.g., when legal investigations were underway) in the two hospitals prohibited the case analysis of all deaths thus limiting comparison between near-misses and deaths. Incomplete documentation and poor storage of patient files were issues that limited the collection and analysis of some variables of interest, such as, e.g., socio-economic status. Routine hospital records do not include detailed socio-demographic data such as socio-economic status or educational level that would have been useful to include in this analysis. These socio-demographic variables are also potential independent factors influencing outcomes. Combining the analysis of medical records with patient interviews could provide richer data that includes detailed socio-demographic variables in future near-miss studies. In addition, only 80% (n = 140) of cases could be matched to women with complications because of low frequency of some maternal conditions (e.g., congestive cardiac failure in pregnancy, epilepsy). Nevertheless, all cases were successfully matched to women without pregnancy related complications. Some of the predictors were very rare or did not occur in the control group. Although we believe that matching is helpful in near miss analyses such data separation will result in large confidence intervals even after Firth correction. Near-miss studies with much larger sample sizes will be required to produce narrower estimates of these large effects. Finally, a number of years have elapsed since the study was conducted. However, we believe that the research methods and findings remain applicable. Other studies indicate that maternal health has not significantly improved since this study was conducted [15].

## Conclusion

Haemorrhage and hypertension were the most common underlying causes of SMO, while anaemia remained an important contributor showing strong association SMOs in these study hospitals. Initiating ANC attendance and having frequent ANC visits were protective against SMOs and need to be encouraged. In addition, differences in maternal outcomes of the two study hospitals despite similar resource inputs suggest that populations from poorer socio-economic backgrounds tend to have poorer outcomes compared to those from better socio-economic backgrounds. Moreover, including a comparison group in near miss studies is useful to quantify relationships between outcomes and factors.

## Supporting information

S1 Table Near-miss criteria. Shows details of the criteria used to identify cases during the study.
(DOCX)

S2 Table Adherence to standards of good practice. Shows the standards of good practice in maternity care measured in this study.
(DOCX)

## References

[1] Bhutta ZA, Das JK, Bahl R, Lawn JE, Salam RA, Paul VK, et al. Can available interventions end preventable deaths in mothers, newborn babies, and stillbirths, and at what cost?. Lancet. 2014;384(9940):347–70. doi: 10.1016/S0140-6736(14)60792-3 24853604

[2] Paxton A, Maine D, Freedman L, Fry D, Lobis S. The evidence for emergency obstetric care. Int J Gynaecol Obstet. 2005;88(2):181–93. doi: 10.1016/j.ijgo.2004.11.026 15694106

[3] Pattinson RC, Buchmann E, Mantel G, Schoon M, Rees H. Can enquiries into severe acute maternal morbidity act as a surrogate for maternal death enquiries?. BJOG. 2003;110(10):889–93. doi: 10.1111/j.1471-0528.2003.03044.x 14550357

[4] Pattinson RC, Hall M. Near misses: a useful adjunct to maternal death enquiries. Br Med Bull. 2003;67:231–43. doi: 10.1093/bmb/ldg007 14711767

[5] Lazzerini M, Richardson S, Ciardelli V, Erenbourg A. Effectiveness of the facility-based maternal near-miss case reviews in improving maternal and newborn quality of care in low-income and middle-income countries: a systematic review. BMJ Open. 2018;8(4):e019787. doi: 10.1136/bmjopen-2017-019787 29674368 PMC5914892

[6] Sayinzoga F, Bijlmakers L, van der Velden K, van Dillen J. Severe maternal outcomes and quality of care at district hospitals in Rwanda- a multicentre prospective case-control study. BMC Preg Childbirth. 2017;17(1):394. doi: 10.1186/s12884-017-1581-4 29178885 PMC5702108

[7] Adeoye IA, Onayade AA, Fatusi AO. Incidence, determinants and perinatal outcomes of near miss maternal morbidity in Ile-Ife Nigeria: a prospective case control study. BMC Preg Childbirth. 2013;13:93. doi: 10.1186/1471-2393-13-93 23587107 PMC3651395

[8] de Moraes APP, Barreto SM, Passos VMA, Golino PS, Costa JE, Vasconcelos MX. Severe maternal morbidity: a case-control study in Maranhao, Brazil. Reprod Health. 2013;10:11. doi: 10.1186/1742-4755-10-11 23399443 PMC3608313

[9] Storeng KT, Drabo S, Ganaba R, Sundby J, Calvert C, Filippi V. Mortality after near-miss obstetric complications in Burkina Faso: medical, social and health-care factors. Bull World Health Organ. 2012;90(6):418-425B. doi: 10.2471/BLT.11.094011 22690031 PMC3370364

[10] Oppong SA, Bakari A, Bell AJ, Bockarie Y, Adu JA, Turpin CA, et al. Incidence, causes and correlates of maternal near-miss morbidity: a multi-centre cross-sectional study. BJOG. 2019;126(6):755–62. doi: 10.1111/1471-0528.15578 30548506 PMC6459711

[11] Soma-Pillay P, Pattinson RC, Langa-Mlambo L, Nkosi BSS, Macdonald AP. Maternal near miss and maternal death in the Pretoria Academic Complex, South Africa: a population-based study. S Afr Med J. 2015;105(7):578–63. doi: 10.7196/SAMJnew.8038 26428756

[12] Mantel GD, Buchmann E, Rees H, Pattinson RC. Severe acute maternal morbidity: a pilot study of a definition for a near-miss. Br J Obstet Gynaecol. 1998;105(9):985–90. doi: 10.1111/j.1471-0528.1998.tb10262.x 9763050

[13] Maswime S, Buchmann E. Causes and avoidable factors in maternal death due to cesarean-related hemorrhage in South Africa. Int J Gynaecol Obstet. 2016;134(3):320–3. doi: 10.1016/j.ijgo.2016.03.013 27352737

[14] National Committee of Confidential Enquiries into Maternal Deaths. Saving mothers 2014-2016: seventh triennial report on confidential enquiries into maternal deaths in South Africa: Short report. Pretoria: National Department of Health; 2017.

[15] NCCEMD. Committee for confidential enquiry into maternal deaths: annual report for 2023. Pretoria: National Department of Health Republic of South Africa; 2023.

[16] NCCEMD. Saving mothers annual report. pretoria: national committe ito confidential enquires of maternal deaths. 2020.

[17] Thwala SBP, Blaauw D, Ssengooba F. “It needs a complete overhaul…” district manager perspectives on the capacity of the health system to support the delivery of emergency obstetric care in an urban South African district. Glob Health Action. 2019;12(1):1642644. doi: 10.1080/16549716.2019.1642644 31362598 PMC6711141

[18] Thwala SBP, Blaauw D, Ssengooba F. Measuring the preparedness of health facilities to deliver emergency obstetric care in a South African district. PLoS One. 2018;13(3):e0194576. doi: 10.1371/journal.pone.0194576 29596431 PMC5875781

[19] van Schaik N. District health barometer 2014/15. Durban: Health Systems Trust; 2015.

[20] Thwala SBP. Health systems factors influencing the delivery of emergency obstetric care in a South African District. Johannesburg: University of the Witwatersrand; 2020.

[21] Statistics South Africa. Census 2011 municipal report. Pretoria: Statistics South Africa; 2012.

[22] WHO. Evaluating the quality of care for severe pregnancy complications: the WHO near-miss approach for maternal health. Geneva: WHO Press; 2011.

[23] Nelissen E, Mduma E, Broerse J, Ersdal H, Evjen-Olsen B, van Roosmalen J, et al. Applicability of the WHO maternal near miss criteria in a low-resource setting. PLoS One. 2013;8(4):e61248. doi: 10.1371/journal.pone.0061248 23613821 PMC3629023

[24] Nelissen EJT, Mduma E, Ersdal HL, Evjen-Olsen B, van Roosmalen JJM, Stekelenburg J. Maternal near miss and mortality in a rural referral hospital in northern Tanzania: a cross-sectional study. BMC Preg Childbirth. 2013;13:141. doi: 10.1186/1471-2393-13-141 23826935 PMC3716905

[25] WHO. Evaluating the quality of care for severe pregnancy complications: the WHO near miss approach for maternal health. 2011.

[26] NDoH. National confidential into maternal deaths audit tool. Pretoria: National Department of Health; 2016.

[27] Firth D. Bias reduction of maximum likelihood estimates. Biometrika. 1993;80(1):27–38.

[28] Greenland S, Mansournia MA, Altman DG. Sparse data bias: a problem hiding in plain sight. BMJ. 2016;352:i1981. doi: 10.1136/bmj.i1981 27121591

[29] Puhr R, Heinze G, Nold M, Lusa L, Geroldinger A. Firth’s logistic regression with rare events: accurate effect estimates and predictions?. Stat Med. 2017;36(14):2302–17. 28295456 10.1002/sim.7273

[30] DoH. Maternal care guidelines South Africa. In: Health. Pretoria: South Africa Government; 2015.

[31] National Department of Health. Maternal care guidelines South Africa: a manual for clinics, community health centers, and hospitals. 2015. https://www.health-e.org.za/wp-content/uploads/2015/11/Maternal-Care-Guidelines-2015_FINAL-21.7.15.pdf

[32] Health ND. Surgical antibiotic prophylaxis recommendations NEML recommendations for medicine amendments (2017 ‐2020). In: South African adult hospital level essential medicines list. Pretoria: South African Government; 2020.

[33] National Committee of Confidential Enquiries into Maternal Deaths. Saving mothers 2011-2013: sixth report on the confidential enquiries into maternal deaths in South Africa. Pretoria: Department of Health; 2014.

[34] Massyn N, Day C, Peer N, Padarath A, Barron P, English R. District health barometer 2013/14. 2014. https://www.health-e.org.za/wp-content/uploads/2014/10/DHB_2013-14.pdf

[35] Wang J, Geng L. Effects of socioeconomic status on physical and psychological health: lifestyle as a mediator. Int J Environ Res Public Health. 2019;16(2):281. doi: 10.3390/ijerph16020281 30669511 PMC6352250

[36] Weimann A, Oni T. A systematised review of the health impact of urban informal settlements and implications for upgrading interventions in South Africa, a rapidly urbanising middle-income country. Int J Environ Res Public Health. 2019;16(19):3608. doi: 10.3390/ijerph16193608 31561522 PMC6801583

[37] Muzindutsi PF, Sekhampu TJ. Determinants of wellbeing in a South African township. Inter J Soc Sci Human Stud. 2014;6(1):1309.

[38] Waterstone M, Bewley S, Wolfe C. Incidence and predictors of severe obstetric morbidity: case-control study. BMJ. 2001;322(7294):1089–93; discussion 1093-4. doi: 10.1136/bmj.322.7294.1089 11337436 PMC31259

[39] Goffman D, Madden RC, Harrison EA, Merkatz IR, Chazotte C. Predictors of maternal mortality and near-miss maternal morbidity. J Perinatol. 2007;27(10):597–601. doi: 10.1038/sj.jp.7211810 17703181

[40] Storeng KT, Drabo S, Ganaba R, Sundby J, Calvert C, Filippi V. Mortality after near-miss obstetric complications in Burkina Faso: medical, social and health-care factors. Bull World Health Organ. 2012;90(6):418-425B. doi: 10.2471/BLT.11.094011 22690031 PMC3370364

[41] Liyew EF, Yalew AW, Afework MF, Essén B. Incidence and causes of maternal near-miss in selected hospitals of Addis Ababa, Ethiopia. PLoS One. 2017;12(6):e0179013. doi: 10.1371/journal.pone.0179013 28586355 PMC5460898

[42] Murphy CM, Murad K, Deane R, Byrne B, Geary MP, McAuliffe FM. Severe maternal morbidity for 2004-2005 in the three Dublin maternity hospitals. Eur J Obstet Gynecol Reprod Biol. 2009;143(1):34–7. doi: 10.1016/j.ejogrb.2008.11.008 19136192

[43] Buchbinder A, Sibai BM, Caritis S, Macpherson C, Hauth J, Lindheimer MD, et al. Adverse perinatal outcomes are significantly higher in severe gestational hypertension than in mild preeclampsia. Am J Obstet Gynecol. 2002;186(1):66–71. doi: 10.1067/mob.2002.120080 11810087

[44] Chappell LC, Enye S, Seed P, Briley AL, Poston L, Shennan AH. Adverse perinatal outcomes and risk factors for preeclampsia in women with chronic hypertension: a prospective study. Hypertension. 2008;51(4):1002–9. doi: 10.1161/HYPERTENSIONAHA.107.107565 18259010

[45] World Health Organisation. WHO recommendations for antenatal care for a positive pregnancy experience. Geneva: World Health Organisation; 2016. 28079998

[46] Moodley J, Pattinson R, Fawcus S, Schoon M, Moran N, Shweni P. The confidential enquiry into maternal deaths in South Africa: a case study. BJOG. 2014. 10.1111/1471-0528.12869 25236634

[47] Abalos E, Cuesta C, Carroli G, Qureshi Z, Widmer M, Vogel J. Pre-eclampsia, eclampsia and adverse maternal and perinatal outcomes: a secondary analysis of the World Health Organization Multicountry Survey on Maternal and Newborn Health. BJOG. 2013;115(10):1265–72. 10.1111/1471-0528.12629 24641531

[48] Lawler J, Osman M, Shelton JA, Yeh J. Population-based analysis of hypertensive disorders in pregnancy. Hypertens Preg. 2007;26(1):67–76. doi: 10.1080/10641950601147945 17454219

[49] You S-H, Cheng P-J, Chung T-T, Kuo C-F, Wu H-M, Chu P-H. Population-based trends and risk factors of early- and late-onset preeclampsia in Taiwan 2001-2014. BMC Preg Childbirth. 2018;18(1):199. doi: 10.1186/s12884-018-1845-7 29855344 PMC5984409

[50] World Health Organisation. WHO recommendations for prevention and treatment of pre-eclampsia and eclampsia. Geneva: World Health Organisation; 2011. 23741776

[51] Pregnancy ACoOaGtTFoHiPHi. Hypertension in pregnancy. Obstet Gynecol. 2013;122:1122–31. 24150027 10.1097/01.AOG.0000437382.03963.88

[52] Abalos E, Duley L, Steyn DW, Gialdini C. Antihypertensive drug therapy for mild to moderate hypertension during pregnancy. Cochrane Database Syst Rev. 2018;10(10):CD002252. doi: 10.1002/14651858.CD002252.pub4 30277556 PMC6517078

[53] Asibong U, Okokon IB, Agan TU, Oku A, Opiah M, Essien EJ, et al. The use of the partograph in labor monitoring: a cross-sectional study among obstetric caregivers in General Hospital, Calabar, Cross River State, Nigeria. Int J Womens Health. 2014;6:873–80. doi: 10.2147/IJWH.S49188 25342920 PMC4206378

[54] Javed I, Bhutta S, Shoaib T. Role of partogram in preventing prolonged labour. J Pak Med Assoc. 2007;57(8):408–11. 17902525

[55] Md Mujibur R, Md Safar A, Kaniz F, Samorendra S, Selina P. The partograph: a practical guide for preventing prolonged labour. Khwaja Yunus Ali Med College J. 2024;14(04):215–9. doi: 10.3329/kyamcj.v14i04.73109

[56] Arunda M, Emmelin A, Asamoah BO. Effectiveness of antenatal care services in reducing neonatal mortality in Kenya: analysis of national survey data. Glob Health Action. 2017;10(1):1328796. doi: 10.1080/16549716.2017.1328796 28621201 PMC5496054

[57] Lambon-Quayefio MP, Owoo NS. Examining the influence of antenatal care visits and skilled delivery on neonatal deaths in Ghana. Appl Health Econ Health Policy. 2014;12(5):511–22. doi: 10.1007/s40258-014-0103-z 24934923

[58] Lagardea M, Palmer N. The impact of user fees on health service utilization in low- and middle-income countries: how strong is the evidence?. Bullet World Health Organ. 2008;86(11). 10.2471/BLT.07.049197 PMC2649541 19030689

[59] Okedo-Alex IN, Akamike IC, Ezeanosike OB, Uneke CJ. Determinants of antenatal care utilisation in sub-Saharan Africa: a systematic review. BMJ Open. 2019;9(10):e031890. doi: 10.1136/bmjopen-2019-031890 31594900 PMC6797296

[60] Downe S, Finlayson K, Tunçalp Ӧ., Metin Gülmezoglu A. What matters to women: a systematic scoping review to identify the processes and outcomes of antenatal care provision that are important to healthy pregnant women. BJOG. 2016;123(4):529–39. doi: 10.1111/1471-0528.13819 26701735

[61] Alanazy W, Brown A. Individual and health systems factors affection antenatal care in Saudi Arabia. BMC Health Serv Res. 2020;20(49). 10.1186/s12913-020-4903-6 PMC6971985 31959162

[62] International A. Struggle for maternal health: barriers to antenatal care in South Africa. Johannesburg: Amnesty International; 2014.

[63] Scholl TO, Hediger ML. Anemia and iron-deficiency anemia: compilation of data on pregnancy outcome. Am J Clin Nutr. 1994;59(2 Suppl):492S-500S. doi: 10.1093/ajcn/59.2.492S 8304287

[64] Lone FW, Qureshi RN, Emmanuel F. Maternal anaemia and its impact on perinatal outcome in a tertiary care hospital in Pakistan. East Mediterr Health J. 2004;10(6):801–7. doi: 10.26719/2004.10.6.801 16335767

[65] Abu-Ouf NM, Jan MM. The impact of maternal iron deficiency and iron deficiency anemia on child’s health. Saudi Med J. 2015;36(2):146–9. doi: 10.15537/smj.2015.2.10289 25719576 PMC4375689

[66] Ubom AE, Begum F, Ramasauskaite D, Nieto-Calvache AJ, Oguttu M, Nunes I, et al. FIGO good practice recommendations on anemia in pregnancy, to reduce the incidence and impact of postpartum hemorrhage (PPH). Int J Gynaecol Obstet. 2025;171(3):993–1007. doi: 10.1002/ijgo.70529 41031541 PMC12640178

[67] Frass KA. Postpartum hemorrhage is related to the hemoglobin levels at labor: observational study. Alexandria J Med. 2015;51(4):333–7. doi: 10.1016/j.ajme.2014.12.002

[68] Ahmed S, Mitra SN, Chowdhury AMR, Camacho LL, Winikoff B, Sloan NL. Community Kangaroo Mother Care: implementation and potential for neonatal survival and health in very low-income settings. J Perinatol. 2011;31(5):361–7. doi: 10.1038/jp.2010.131 21311502

[69] Carroli G, Villar J, Piaggio G, Khan-Neelofur D, Gülmezoglu M, Mugford M, et al. WHO systematic review of randomised controlled trials of routine antenatal care. Lancet. 2001;357(9268):1565–70. doi: 10.1016/S0140-6736(00)04723-1 11377643

[70] Maswime S, Buchmann E. Why women bleed and how they are saved: a cross-sectional study of caesarean section near-miss morbidity. BMC Preg Childbirth. 2017;17(15). 10.1186/s12884-016-1182-7 PMC5223297 28068945

[71] Daru J, Zamora J, Fernández-Félix BM, Vogel J, Oladapo OT, Morisaki N, et al. Risk of maternal mortality in women with severe anaemia during pregnancy and post partum: a multilevel analysis. Lancet Glob Health. 2018;6(5):e548–54. doi: 10.1016/S2214-109X(18)30078-0 29571592

[72] Health DoH. Guidelines for maternity care in South Africa: a manual for clinics, community health centers, and district hospitals. 3rd edition ed. Pretoria: National Department of Health; 2007.

