# Supplementary material for: Clinical factors associated with severe maternal outcomes in two South African hospitals: A case-control study
Source: PLoS One. 2026 Apr 15;21(4):e0346119. doi: 10.1371/journal.pone.0346119 (PMC13082711; doi:10.1371/journal.pone.0346119)
Supplement: S1 Table — Shows details of the criteria used to identify cases during the study. (DOCX) [file pone.0346119.s001.docx]

**S1 Table: Near-miss criteria**

| **WHO criteria** | **Adapted criteria used in the study** |
| --- | --- |
| **Clinical criteria** | |
| Acute cyanosis | Acute cyanosis |
| Gasping | Gasping |
| Respiratory rate >40<6 per minute | Respiratory rate >40<6 per minute |
| Shock | Shock |
| Oliguria non-responsive to fluids or diurectics | Oliguria non-responsive to fluids or diurectics |
| Failure to form clots | Failure to form clots |
| Loss of consciousness>12 hours | Loss of consciousness>12 hours |
| Cardiac arrest | Cardiac arrest |
| Stroke | Stroke |
| Uncontrollable fit/ total paralysis | Uncontrollable fit/ total paralysis |
| Jaundice in the presence of pre-eclampsia | Jaundice in the presence of pre-eclampsia |
| **Laboratory based criteria** | |
| Oxygen saturation <90% >=60 minutes | Oxygen saturation <90%>=60 |
| PaO2/FiO2 <200mmHg |  |
| Creatinine >=300umols/l or = 3.5 mg/dl |  |
| Bilirubin >100umols/l or >6.0mg/dl |  |
| pH < 7.1 |  |
| Lactate > 5 mEq/ml |  |
| Acute thrombocytopenia (<50,000 platelets/ ml) | Acute thrombocytopenia (<50,000 platelets/ml) |
| Loss of consciousness and ketoacids in urine |  |
| **Severe maternal complications/ Disease based** | |
|  | Eclampsia |
|  | Uterine rupture |
| **Management based criteria** | |
| Blood transfusion >=5litres | Blood transfusion>=1 litres |
| Hysterectomy | Hysterectomy |
| Dialysis | Dialysis |
|  | Admission into ICU |
