# Supplementary material for: Clinical factors associated with severe maternal outcomes in two South African hospitals: A case-control study
Source: PLoS One. 2026 Apr 15;21(4):e0346119. doi: 10.1371/journal.pone.0346119 (PMC13082711; doi:10.1371/journal.pone.0346119)
Supplement: S2 Table — Shows the standards of good practice in maternity care measured in this study. (DOCX) [file pone.0346119.s002.docx]

**S2 Table: Adherence to standards of good practice**

| **Category** | **Indicator** | **Level**  **Achieved** |
| --- | --- | --- |
| **Routine Care (n=490)** | ANC booked | 462 (94.29%) |
|  | Oxytocin given in 3^rd^ stage of labour | 478 (98.6%) |
|  | Syntometrine in 3^rd^ stage of labour | 7(1.4%) |
|  | Tested for syphilis | 490 (100.0%) |
|  | HIV status Target | 490 (100.0%) |
|  | Partogram used | 217(60.96%) |
| **C/S (n-203)** | Prophylactic antibiotics | 203(100.0%) |
| **HIV +ve (n=145)** | PMTCT given | 139(95.86%) |
| **PIH (n=87)** | Mg_2_S0_4_ given | 50(66.67%) |
| **Obstetric haemorrhage (n=75)** | Oxytocin | 54 (72.0%) |
|  | Ergometrine | 6 (8.0%) |
|  | Misoprostol | 21 (28.0%) |
